# Atmosphere-Controlled Synthesis of Hierarchical Cu/Cu_2_O/CuO Microtube Architectures Decorated with High-Density CuO Nanowires from Recycled E‑Waste

**DOI:** 10.1021/acs.langmuir.5c06745

**Published:** 2026-03-20

**Authors:** Suzilene V. Santos, Crystian W. C. Silva, Pedro H. Britto-Costa, Thiago Lopes, Sávio F. Silva, Cleidilane S. Costa, Larissa Otubo, C. M. Rivaldo-Gómez, Artur W. Carbonari, Gabriel A. Cabrera-Pasca

**Affiliations:** † 176839 Universidade Federal do Pará (UFPA), Abaetetuba, Pará 09210-580, Brazil; ‡ 119500 Instituto de Pesquisas Energéticas e Nucleares, Comissão Nacional de Energia Nuclear, IPEN-CNEN/SP, São Paulo, SP 05508-000, Brazil; § Research Centre for Greenhouse Gas Innovation (RCGI), 28133 University of São Paulo, Escola Politécnica, Av. Professor Mello Moraes, 2231−Cidade Universitária, São Paulo, SP 05508-030, Brazil; ∥ Research Center for Greenhouse Gas Innovation (RCGI), University of São Paulo, Escola Politécnica, Av. Professor Mello Moraes, 2231−Cidade Universitária, São Paulo, SP 05508-030, Brazil; ⊥ Research Center for Greenhouse Gas Innovation (RCGI), University of São Paulo, Escola Politécnica, Av. Professor Mello Moraes, 2231−Cidade Universitária, São Paulo, SP 05508-030, Brazil; # European Organization for Nuclear Research, Esplanade des Particules 1, 1217 Meyrin, Switzerland

## Abstract

The design of hierarchical copper oxide microstructures with tailored nanomorphologies is essential for next-generation electrocatalytic, sensing, and nanoelectronic applications. Here, we report an atmosphere-controlled thermal oxidation route for the sustainable synthesis of hollow Cu/Cu_2_O/CuO microtubes decorated with a dense array of vertically aligned CuO nanowires. The synthesis uses recycled copper microwires from electronic waste (e-waste) coated with a polyurethane (PU) polymer. Comparative analysis under ambient air and high-purity synthetic air reveals that the thermal degradation of the polymeric coating forms a carbon-rich layer, crucial for regulating asymmetric cation transport and inducing mass transport that drives void formation via the Kirkendall effect. This mechanism transforms the solid microwire into a concentric hollow microtube structure. Critically, oxidation under synthetic air promotes the extensive growth of long CuO nanowires (up to 20 μm), guided by anisotropic diffusion along twin boundaries and sustained by a strong chemical potential gradient. These findings establish atmosphere control as a powerful strategy to fine-tune the multiscale architecture of sustainable metal oxide nanostructures from e-waste precursors, opening pathways for the scalable production of multifunctional materials.

## Graphical abstract

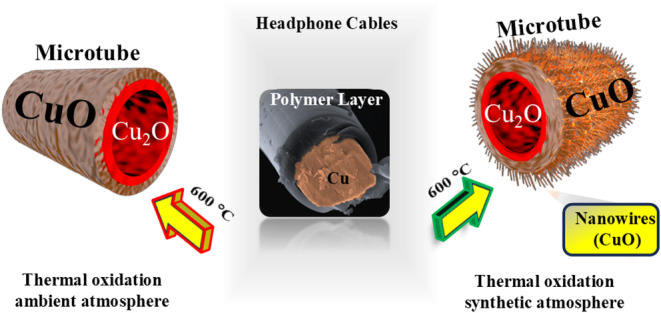

## Introduction

The development of novel strategies for the synthesis of hollow micro- and nanostructured materials has attracted considerable attention due to their relevance in clean and sustainable technologies. Hollow architectures, such as nanospheres, nanotubes, nanorods, and microtubes, offer a high surface area, low density, and enhanced chemical reactivity, making them attractive for applications in drug delivery, energy storage, gas sensing, solar cells, photocatalysis, and optoelectronics. Among synthetic strategies, thermal oxidation is an attractive, simple, and scalable template-free technique for fabricating hierarchical metal oxide nanostructures.^1–5^

Thermal oxidation is governed by the diffusion of oxygen species and their subsequent chemical reaction with the metallic surface to form oxide layers. Tubular oxides of transition metals, such as ZnO, TiO_2_, and Fe_2_O_3_, have been extensively explored owing to their diverse physical and chemical properties, including p-type conductivity, high chemical stability, visible-light absorption, and potential for energy conversion applications.^5–10^ A notable example was reported by Rivaldo-Gómez et al.,^5^ who demonstrated the formation of TiO_2_ microtubes via a thermal oxidation process combined with an electric-current-induced phase transition, highlighting an enhanced ionic diffusion phenomenon. Furthermore, the development of hierarchical morphologies from these systems, such as the functionalization and growth of nanostructures on microtube surfaces, enables the construction of multifunctional architectures, expanding the application prospects of such hybrid systems.^11–14^ These architectures allow fine control over the geometry, crystallographic orientation, and surface properties, which are crucial for optimizing electrical, magnetic, and optical behavior. For example, TiO_2_ microtubes decorated with CsPbBr_3_ nanocrystals exhibited enhanced charge transport, while In_2_O_3_ microtubes functionalized with Ag nanoparticles showed high sensitivity for NO_2_ detection.^15^ Although the formation of ZnO and TiO_2_ microtubes from the thermal oxidation of Zn and Ti microwires is well established, analogous attempts with Ni and Cu microwires have not yielded hollow structures.^16,17^ This highlights the need for further investigations to elucidate the conditions that favor such transformations, particularly in Cu-based systems due to the abundance and applicability of this metal.^6,18^

Beyond the abundance and low cost of the metallic precursor (Cu), the synthesis of copper oxide microtubes (specifically cuprous oxide (Cu_2_O) and cupric oxide (CuO)) is particularly appealing due to their semiconducting nature. These p-type, nontoxic, low-bandgap semiconductors exhibit remarkable optical, magnetic, and electronic properties, including high photoelectric conversion efficiency and complex phase behavior.^2,19,20^ In the context of the circular economy and green nanotechnology, developing innovative processing methods for recycling e-waste—specifically copper microwires from components like headphone cables—is a global imperative.^18,21,22^ Copper recycling not only reduces energy consumption but also strengthens the circular economy. However, to make this recycling effective, innovative processing methods capable of generating higher value-added structures from waste streams are required.^23^ In this regard, the thermal oxidation of copper microwires recovered from electronic components can be envisioned as a promising pathway for the fabrication of functional micro- and nanostructures.^24–26^ This enables the reuse of these materials in other applications, such as efficient catalysts for hydrogen production via the hydrogen evolution reaction (HER), photocatalysts for water purification, and in energy storage devices.^2,27–29^

In this work, we report the atmosphere-controlled growth of hierarchical Cu/Cu_2_O/CuO microtubes decorated with CuO nanowires, obtained through the thermal oxidation of recycled Cu microwires coated with a polyurethane (PU) polymer layer.^30,31^ The polymer plays a dual role: it acts as a protective coating during the initial heating stage and subsequently decomposes into a carbonaceous barrier that regulates ionic diffusion, in a manner similar to processes employed in the fabrication of hollow nanostructures.^15^ Oxidation processes under ambient air and flowing synthetic air revealed distinct morphological evolutions with the symmetry of the microtubes and the density of nanowires being strongly dependent on the oxidation environment. A particularly interesting result is the observation of enhanced ionic diffusion and void formation via the Kirkendall effect,^32,33^ which facilitates the transformation of solid Cu microwires into hollow oxide microtubes within relatively short reaction times. Under synthetic air, more intense oxidation conditions led to the growth of long CuO nanowires (up to 20 μm), favored by anisotropic diffusion along twin boundaries and strong chemical potential gradients. The resulting multiscale structures were comprehensively characterized by X-ray diffraction (XRD), scanning and transmission electron microscopy (SEM and TEM-HRTEM), and Raman spectroscopy to elucidate their crystallographic phases, morphological evolution, and the chemical nature of the surface oxides. This study presents a sustainable and reproducible route for producing Cu-based hierarchical structures from electronic waste, offering valuable insights for the design of multifunctional materials for sensing, catalysis, and nanoelectronics applications.

## Results and Discussion

### Formation of Hierarchical Cu/Cu_2_O/CuO Microtubes under Ambient Atmosphere

Hollow copper oxide microtubes and related microstructures decorated with nanowires were synthesized by the thermal oxidation of metallic copper (Cu) microwires at elevated temperatures. Figure 1(a–c) presents SEM images of a cross-section of a recycled copper microwire, initially coated with a polymeric barrier (polyurethane (PU)) derived from headphone cables. Figure S1 in the Supporting Information shows the characterization of the microwire by Fourier transform infrared spectroscopy (FTIR). The polymeric layer displays a thickness of approximately 5.25 μm, while the Cu metallic core has a diameter of approximately 40 μm.

**Figure 1:**
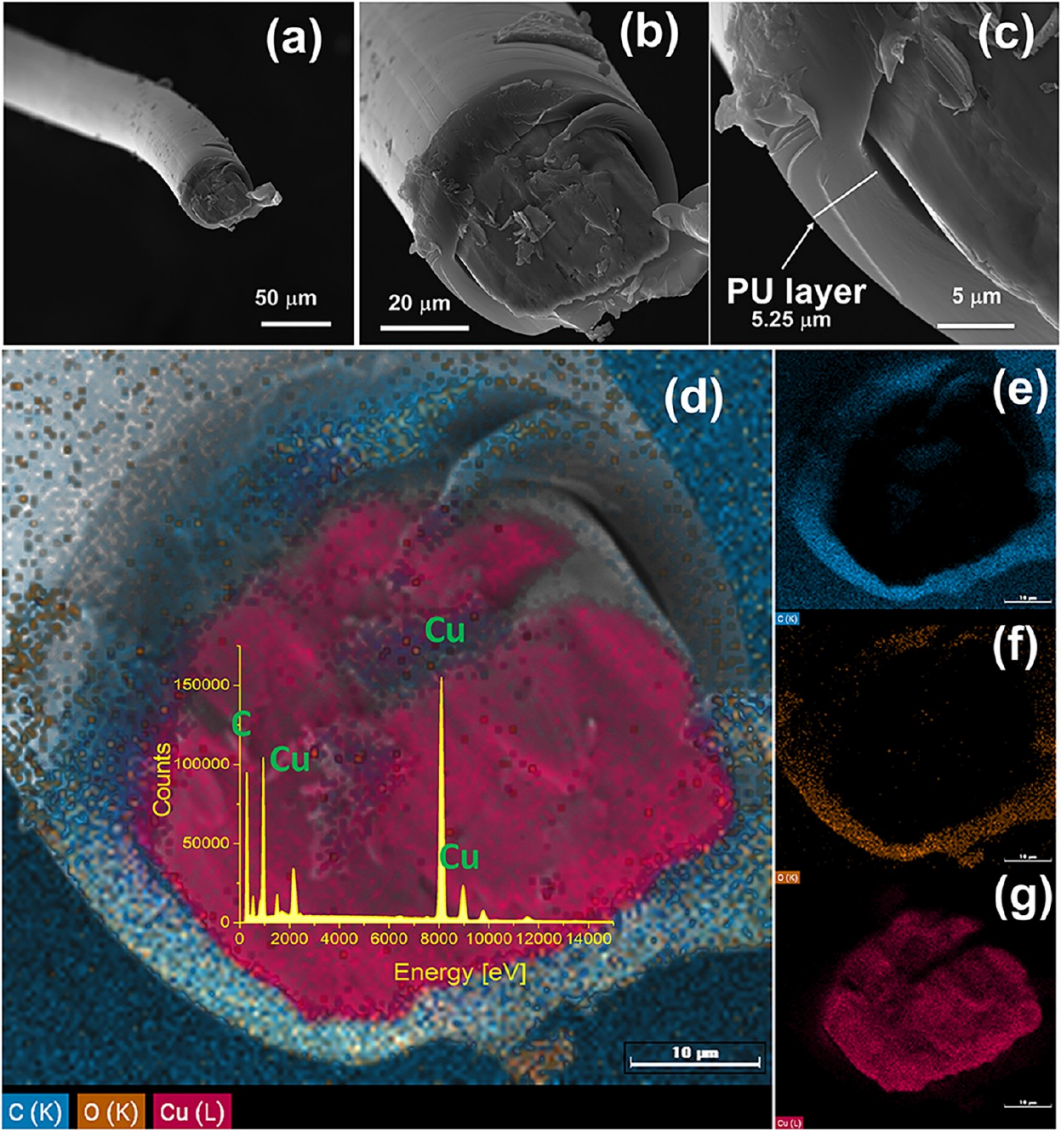
(a, b) Scanning electron microscopy (SEM) images of copper microwires coated with a protective polymer layer, including a cross-sectional view. The inset highlights the metallic Cu core with an average diameter of 40 μm. (c) Details of the polymeric coating, approximately 5.25 μm thick, surrounding the Cu core. (d–g) EDS elemental mapping of the cross-section: blue indicates carbon (C), orange corresponds to oxygen (O), both related to the polymer layer, and red denotes the metallic copper (Cu) core at the center of the wire.

During the oxidation process, the polyurethane (PU) coating was intentionally retained. Commonly used in electronic cables, this polymer acts as a barrier to free ionic and electronic diffusion, providing corrosion resistance, electrical insulation, and mechanical stability. EDS mapping (Figure 1(d–g)) confirms the presence of carbon and oxygen associated with the PU layer. At approximately 400 °C, a thin surface layer rich in residual carbon is observed, as inferred from the TGA data and microstructural observations. This carbonaceous layer acts as a physical diffusion barrier, limiting oxygen ingress and thereby suppressing oxidation of the copper core. As a result, surface structures such as Cu@C or CuO@C are formed, without the development of CuO nanowires, although their formation is commonly reported for planar copper substrates at similar temperatures.^34,35^

Importantly, the presence of the polymeric coating also plays a critical role in the formation of voids between the metallic Cu core and the oxidized shell. This phenomenon can be attributed to a Kirkendall-type mechanism, often reported in hollow nanostructures such as Co_3_O_4_, SnO_2_, and CuO.^32,36–38^ In this case, the degraded PU layer limits oxygen diffusion inward while still allowing the migration of Cu^+^/Cu^2+^ migration outward. As the flux of Cu atoms/ions is higher than that of oxygen, vacancies accumulate inside the structure and coalesce into voids. This imbalance is described by

1 $$J_{\mathrm{void}}=J_{\mathrm{Cu}}-J_{O}$$

where:

- *J*_Cu_ is the outward flux of Cu,
- *J*_O_ the inward flux of oxygen,
- *J*_void_ is the net vacancy flux toward the interior, favoring void coalescence when *J*_Cu_ > *J*_O_.

This mechanism aligns with previous findings by Jong Min Won et al. and Jing Hu et al. supporting the formation of Cu@C and CuO@C structures and reinforcing the hypothesis that the diffusion regime, modulated by the decomposing PU layer, is a key to the resulting morphology.^38,39^

### Controlled Oxidation Time for Microtube Formation

To better understand the oxidation dynamics and hollowing mechanism in an ambient atmosphere, Set 1 (S_1_) samples were divided into two experimental subgroups: (a) S_1A_: Samples were oxidized at 600 °C for 1 h, followed by furnace cooling; and (b) S_1B_: Samples were oxidized at the same temperature (600 °C) for an extended period of 6 h.

SEM analysis of S_1A_ revealed a core–shell structure with a metallic Cu core surrounded by concentric layers of Cu_2_O and CuO. The surface exhibited a porous and granular morphology, with a low density of nanowires (see Figure S2 in the Supporting Information).

For S_1B_, SEM analysis (Figure 2(a–e)) showed that the longer exposure led to the formation of fully developed, symmetric hollow CuO microtubes with good structural integrity and no visible fractures. Residual metallic Cu was occasionally observed within the polycrystalline tubular walls, as shown in Figure 2(b,e). Figure 2(f) presents a magnified cross-section of a representative microtube, highlighting its layered internal structure, resembling those reported by Košiček et al.^40^ The porous inner region shows delamination of Cu/Cu_2_O layers from a dense columnar Cu_2_O interface, especially upon cooling (suggesting dynamic oxidation stages). According to Koiek et al., early-stage Cu_2_O grains serve as entry points for Cu diffusion, facilitating outward migration from the metallic core toward the oxide layers. In this study, this behavior is consistently observed across samples: the interface begins with fine Cu_2_O grains, progresses into columnar growth, and culminates in a dense outer Cu_2_O layer, followed by a porous CuO surface. Figure 2(g–h) also reveals isolated CuO nanowires (∼10 μm in length) emerging from the outer surface of the microtubes.

**Figure 2:**
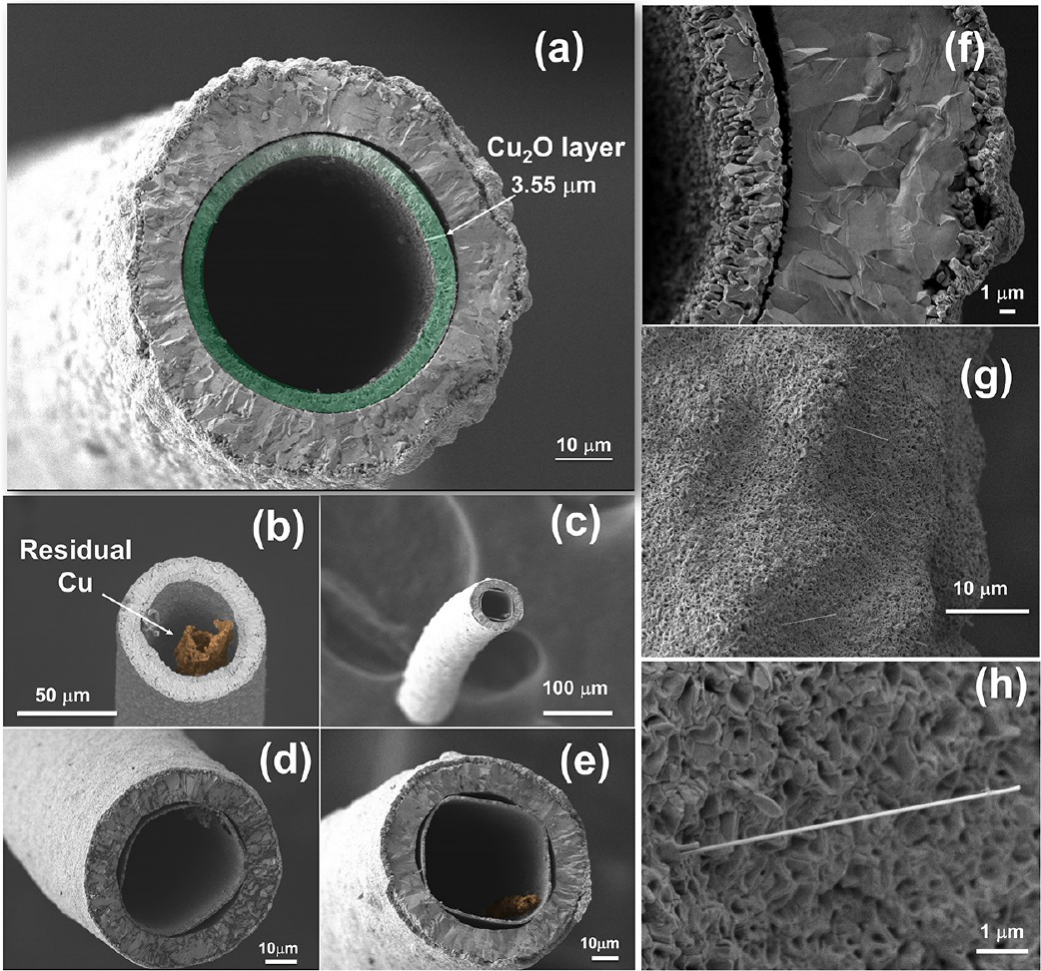
(a–e) SEM images of copper oxide microtubes obtained after thermal oxidation at 600 °C for 6 h in a conventional muffle furnace, followed by cooling in air. Panels (b) and (e) show the presence of residual metallic Cu. (f) Cross-sectional view of a representative microtube, revealing concentric layers with distinct morphologies. (g–h) Surface details of the outer wall, highlighting isolated CuO nanowires protruding from the microtube surface.

### Structural and Compositional Analysis: XRD, EDS, and Raman Spectroscopy

Figure 3 summarizes the structural characterization of the samples by XRD (Rietveld refinement). Figure 3(a) shows the XRD pattern of the pristine Cu wire, revealing the characteristic (111), (200), and (220) reflections of face-centered cubic (FCC) Cu, space group *Fm*3*m*. Figure 3(b) presents the XRD data after 1 h at 600 °C, with reflections corresponding to both Cu (*Fm*3*m*) and Cu_2_O (*Pn*3*m*). The sample retained approximately 91% metallic Cu, confirming partial oxidation. Figure 3(c–d) shows data from samples oxidized for 6 h, analyzed in two forms: (c) powder (crushed microtubes) and (d) intact tubular structures. The powdered sample displays Cu, Cu_2_O, and CuO phases, while the tubular sample shows only Cu_2_O and CuO, suggesting that metallic Cu remains localized in internal regions and below the XRD detection threshold when unbroken. Reflections attributed to CuO match planes such as (110), (002), (111), (2̅02), (020), and (311), corresponding to its monoclinic structure (space group *C*2/*c*).

**Figure 3:**
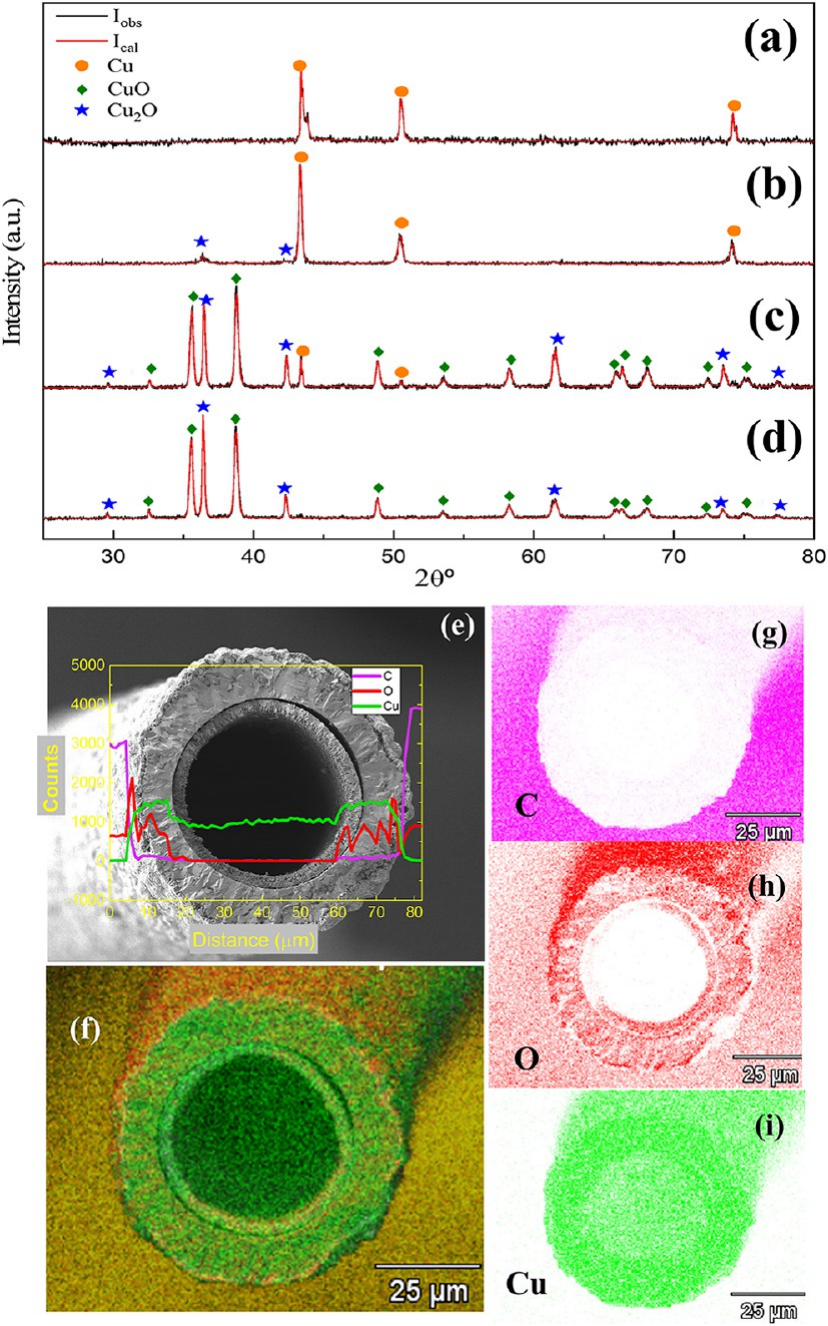
(a) X-ray diffraction (XRD) pattern of the pristine Cu microwire coated with polymer. (b) XRD of the microwire after thermal treatment at 400 °C in air, showing partial oxidation. (c) XRD of the powder (crushed microtubes) sample after full oxidation. The markers below each pattern correspond to the expected Bragg reflections for Cu (*Fm*3̅*m*), Cu_2_O (*Pn*3̅*m*), and CuO (*C*2/*c*) crystal phases. (d) XRD of the tubular structure after full oxidation. The markers now correspond to the expected Bragg reflections for the Cu_2_O (*Pn*3̅*m*) and CuO (*C*2/*c*) crystal phases. No Cu (*Fm*3̅*m*) phase was observed. (e) EDS line-scan across the tube wall, showing a higher oxygen concentration at the outer layer relative to the inner region. (f–i) Elemental mapping of Cu, O, and residual C, respectively, consistent with the formation of a Cu/Cu_2_O/CuO layered structure.

EDS mapping and line-scan profiling of microtube cross sections reveal a concentric trilayer structure, composed of: an inner metallic Cu shell (as confirmed by powdered XRD), an intermediate Cu_2_O layer, an outer porous CuO layer, and traces of residual carbon from the PU coating degradation. Figure 3(e) displays the overall tubular morphology, showing a porous surface consistent with advanced oxidation. The line-scan confirms a low carbon content at the surface and distinct elemental distributions across the layers, validating the Cu/Cu_2_O/CuO hierarchical structure. The elemental distribution was observed in Figure 3(f,i), where the EDS maps show color-coded elemental distribution: green (Cu) in the inner layer, red (O) across oxidized regions, and purple (C) indicating traces of residual PU combustion. Additionally, the unequivocal presence of monoclinic CuO was also confirmed by Raman spectroscopy (see Figure S3 in the Supporting Information), corroborating the findings obtained from XRD and EDS.

### Thermal Oxidation under Flowing Synthetic Air

Maintaining the same heating rates used for Set 1, the copper microwires were oxidized under flowing synthetic air (≈21 vol % O_2_), with constant gas flow (100 mL·min^–1^) monitored by thermogravimetric analysis (TGA) as a function of time and temperature. These samples were designated as Set 2 (S_2_), and they were divided into two subgroups, S_2A_ and S_2B_. For the S_2A_ group, the samples were subjected to oxidation at 600 °C for 5 min, followed by natural cooling under a flow of 100 mL·min^–1^ of synthetic air. The SEM micrographs obtained for this group show the partial formation of microtubes (see Figure S4 in the Supporting Information).

For S_2B_, the microwires were held at 600 °C for 6 h, also under synthetic air flow, with in situ TGA monitoring (see Figure 4). The TGA curve reveals a small initial mass loss (∼10%) attributed to the degradation of the polyurethane (PU) coating, followed by a mass gain of ∼24%, indicating progressive oxidation of the metallic copper core. After the mixture was cooled, SEM images revealed the formation of copper oxide microtubes with a high density of nanowires on their surfaces.

**Figure 4:**
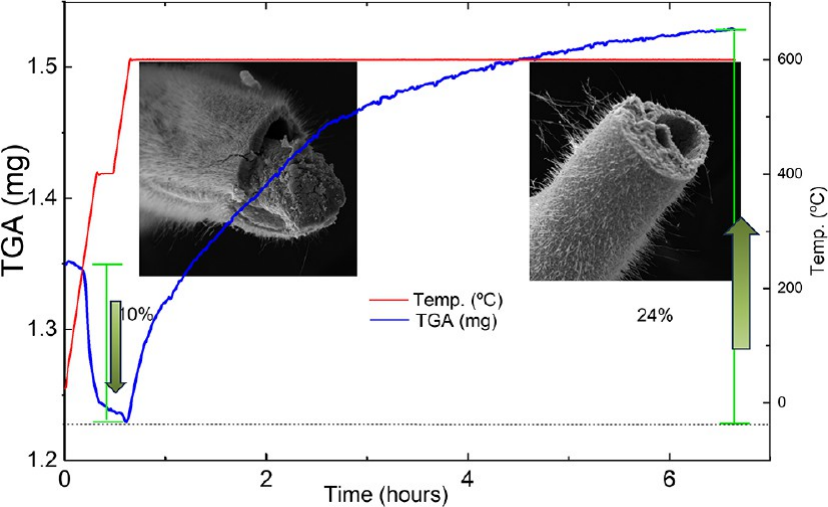
Transformation of metallic Cu microwires into hollow microtubes during the thermal oxidation process under flowing synthetic air (flow rate: 100 mL·min^–1^), monitored by thermogravimetric analysis (TGA). The blue curve shows the mass variation over time, while the red curve indicates the applied heating rate. An initial mass loss of ∼10% is observed, attributed to the thermal degradation of the polymeric coating. Above ∼400 °C, a continuous mass gain of approximately 24% occurs, consistent with the progressive formation of copper oxide layers (Cu_2_O and CuO) through oxidation of the metallic Cu core. SEM micrographs depict distinct morphological stages throughout the oxidation process from the initial microwire structure to the final hollow microtube architecture.

The microtubes synthesized under synthetic air exhibited a distinctly asymmetric tubular morphology, as shown in Figure 5(a–d), in sharp contrast to the symmetric and concentric architecture observed for the Set 1 samples oxidized under ambient air. The internal structure displays well-defined columnar growth of the intermediate Cu_2_O layer that gradually transitions into a denser outer CuO shell. This outer region is further decorated by a high-density array of CuO nanowires, as evidenced in Figure 5(e–f).

**Figure 5:**
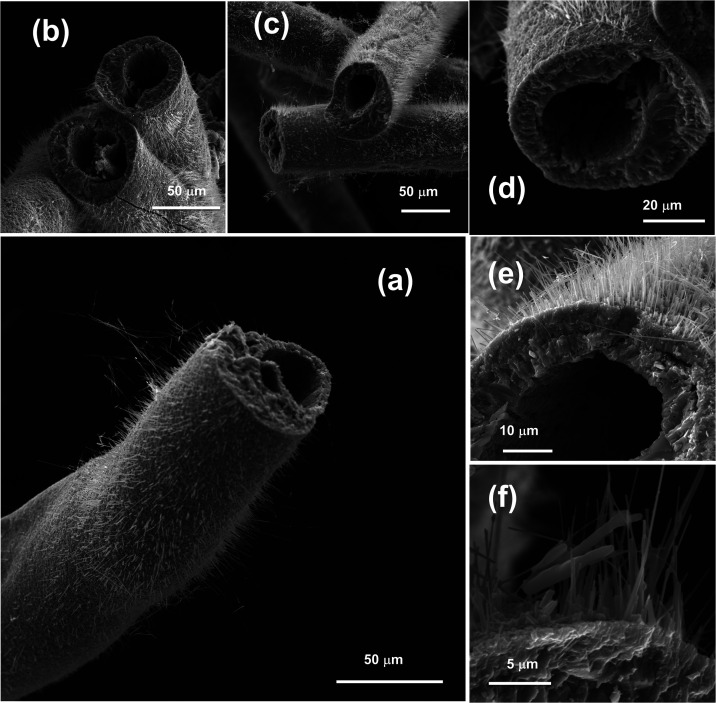
SEM images of Cu_2_O/CuO hollow microtubes synthesized under flowing synthetic air (100 mL·min^–1^) at 600 °C for 6 h; oxidation performed in a furnace monitored by TGA/DTA. (a–c) Overview microtube images showing asymmetric and irregular hollow tubular morphologies. (d) High-magnification image revealing internal surface roughness and partial wall collapse. (e) Cross-sectional view of a microtube displaying a compact inner Cu_2_O layer and the initial formation of vertically oriented CuO nanowires on the outer surface. (f) Detailed view of the external surface showing CuO nanowires exceeding 10 μm in length, with evident coalescence at their bases.

SEM analysis revealed nanowires exceeding 10 μm in length, reaching up to 20 μm in some instances. X-ray diffraction confirmed that the resulting phase is predominantly monoclinic CuO (95.3%), with a minor Cu_2_O contribution (4.7%). In contrast to the Set S1 samples, where nanowires were scarce or absent, those treated under synthetic air exhibited extensive nanowire growth despite identical thermal conditions. This observation underscores the decisive influence of the oxidation atmosphere and the oxygen partial pressure on the resulting hierarchical morphology.

A recurrent morphological feature among these samples is the presence of thickened nanowire bases, particularly near the outer microtube wall (Figure 5f). These basal regions often comprise bundles of closely packed nanowires with variable diameters and clear indications of coalescence and crystal twinning. This structural motif is consistent with the observations of Košiček et al.,^40–42^ who reported that the lower segments of CuO nanowires frequently contain multiple twin boundaries and appear thicker due to selective growth continuation in specific twinned crystallites. Such behavior suggests that anisotropic nucleation and early-stage growth along preferential twin planes—facilitated by high oxygen availability and the presence of crystalline defects—govern the formation of the hierarchical CuO nanowire morphology.

Collectively, these results provide compelling evidence that twin-boundary-assisted growth, in combination with a controlled oxidation environment, synergistically promotes the formation of vertically aligned, high-aspect-ratio CuO nanowires. The presence of twin boundaries likely reduces the energetic barrier for axial elongation, thereby facilitating the emergence of elongated nanostructures protruding radially from the CuO microtube surface.

Further insights were obtained through high-resolution transmission electron microscopy (HRTEM) of isolated nanowires, as displayed in Figure 6. These analyses revealed central planar defects, unambiguously identified as twin boundaries, that are consistent with the mechanisms of directional crystal growth. Nanowire bases frequently appeared thicker and irregular, supporting a model involving multigrain coalescence and defect-guided elongation.

**Figure 6:**
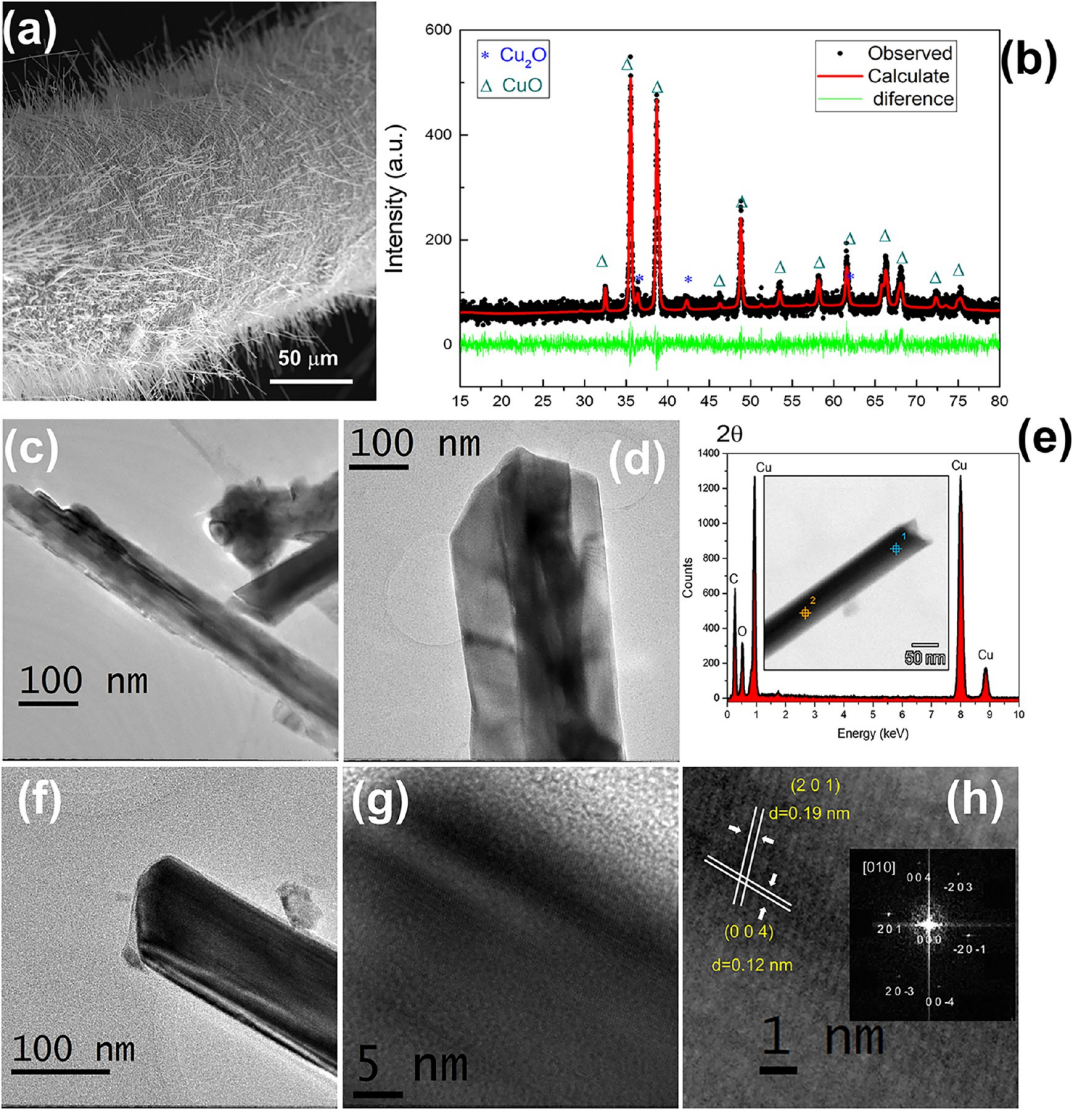
(a) SEM image of the outer surface of a CuO microtube showing a high density of vertically aligned CuO nanowires. (b) XRD pattern of the microtube surface, confirming the predominance of monoclinic CuO with minor Cu_2_O contributions. (c–f) High-resolution transmission electron microscopy (HRTEM) images of isolated nanowires, exhibiting varying diameters due to nanowire coalescence and the presence of twin boundaries. (g, h) Magnified HRTEM images showing lattice fringes corresponding to the CuO (201) crystal plane with an interplanar spacing of *d* = 0.19 nm.

The surface of the samples exhibits a high density of CuO nanowires, with an average length of approximately 9.1 μm and a standard deviation of 6.4 μm, following a γ-type distribution (see Figure 7a). Hierarchical clustering analysis of the length distribution, employing the Ward method, reveals that the sample does not exhibit a monomodal behavior but rather consists of three statistically distinct nanowire populations. These clusters are centered at approximately 4.3 μm (48.9%), 10.9 μm (38.3%), and 22.3 μm (12.8%), as shown in Figure 7b, indicating a clear predominance of short and intermediate-length nanowires, in good agreement with previous reports by Shao-Liang Cheng and Košiček et al.^40,41^ Although most nanowires present lengths below 10 μm, a small fraction can reach and even exceed 30 μm. At this stage, the copper core is still partially preserved, and no hollow tubular structure has yet been observed. The oxide layers exhibit distinctive morphologies, with columnar Cu_2_O grains and porous regions associated with CuO formation.

**Figure 7:**
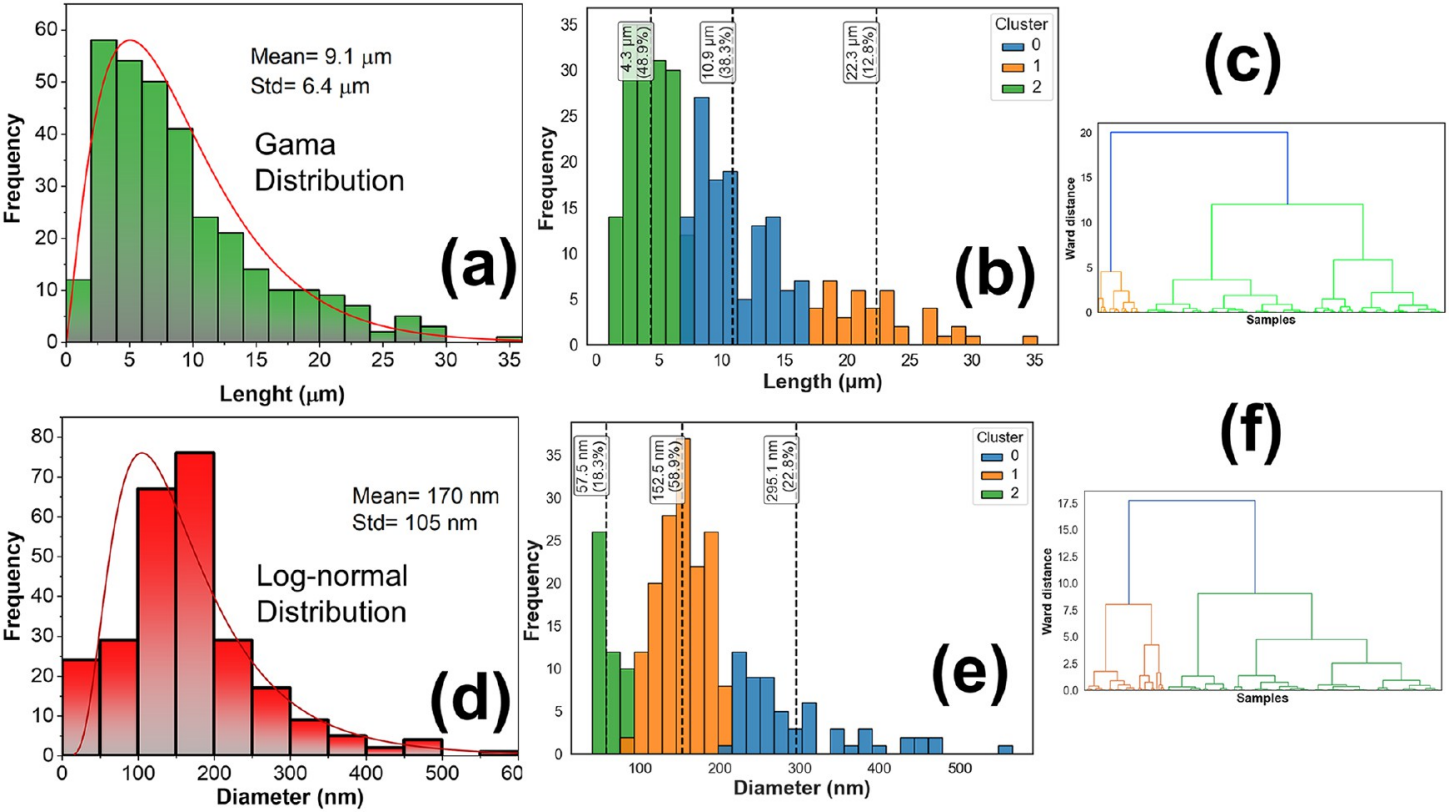
Descriptive statistics of the nanowire lengths and diameters obtained from SEM and TEM analyses, respectively. (a) Histogram of length distribution. (b, c) Hierarchical clustering of nanowire lengths. (d) Histogram of diameter distribution. (e, f) Hierarchical clustering of nanowire diameters.

Based on the TEM/HRTEM analyses, it was observed that the diameter distribution of nanowires exhibits asymmetric behavior and is well described by a log-normal function, with a mean value of approximately 170 nm and a standard deviation of 105 nm (see Figure 7d), indicating a significantly polydisperse system. Hierarchical clustering analysis of diameter distribution using the Ward method reveals that the sample is composed of three statistically distinct populations, centered at approximately 57.5 (18.3%), 152.5 (58.9%), and 295.1 nm (22.8%), as can be seen in Figure 7e. The intermediate population is predominant and can be associated with a stable growth regime, whereas the thinner and thicker nanowires can be correlated, respectively, with early stages of nucleation/growth and with regions where pronounced growth occurs, likely involving a coalescence mechanism. In addition, HRTEM images show well-resolved lattice fringes displaying the (201) and (004) CuO planes, indexed with respect to the [010] zone axis. These results confirm the presence of a hierarchical structure, with CuO predominantly located in the outer regions of the nanowires. The larger diameters were attributed to coalesced domains formed from dense twinning, as also shown in Figure 5f.

Complementary XRD measurements of powdered microtubes confirmed the absence of residual metallic Cu, indicating the complete oxidation of the original Cu core. This finding further supports the formation of asymmetric Cu_2_O/CuO/CuO nanowire architectures, with the surface nanowires emerging as a consequence of localized diffusion gradients and twin-facilitated crystal growth mechanisms favored under synthetic air conditions.

It should be noted that the oxidation experiments performed under ambient air and synthetic air were conducted in distinct setups, involving differences in the gas flow regime, furnace geometry, humidity, and sample configuration. As a result, the observed oxidation behavior reflects the combined influence of gas composition and transport conditions rather than the gas composition alone.

In addition to oxygen availability, ambient humidity is expected to influence the oxidation pathway and the resulting morphology. Under ambient air conditions, the presence of water vapor can modify surface reaction kinetics by promoting hydroxylated surface species, which may enhance surface diffusion and favor more uniform oxide growth. Such conditions can suppress localized nanowire nucleation by reducing the oxidation asymmetry and stabilizing continuous oxide layers. In contrast, the dry synthetic air environment minimizes the contribution of water-mediated surface processes, thereby enhancing the directional mass transport and oxidation asymmetry, which are conducive to nanowire formation. While the present study does not independently decouple the effects of oxygen partial pressure and humidity, the observed morphological differences are consistent with the combined influence of reduced humidity and controlled oxygen flux in the synthetic air configuration.

### Nanowire Growth Mechanism in Synthetic Atmosphere

The growth of copper oxide (CuO) nanowires is a well-established phenomenon during the thermal oxidation of copper, frequently attributed to a stress-driven mechanism.^40,43,44^ The external surface of the microtubes, composed predominantly of CuO, acts as an active site for vertical nanowire growth with morphology and density strongly influenced by atmospheric and structural factors, particularly the formation of twin defects. The following

mechanisms are proposed: (a) Cu^+^/Cu^2+^ diffusion through twin boundaries: CuO grains containing symmetrically joined crystallites (twins) serve as preferred pathways for unidirectional Cu ion migration. This anisotropic diffusion reduces the energy barrier for atomic transport and promotes axial nanowire growth. (b) Surface diffusion of the Cu^+^ species: In parallel, Cu ions migrate along the CuO surface, where they encounter atmospheric oxygen and nucleate nanowires. This mechanism becomes dominant in oxygen-rich environments, where Cu^+^ oxidation at the surface is continuous. (c) Chemical potential gradient: The hierarchical structure (Cu/Cu_2_O/CuO) generates a chemical potential gradient from the interior (Cu-rich) to the surface (O_2_-rich), particularly under synthetic air, which sustains a continuous Cu ion flux. This gradient is essential to drive microtube formation under ambient conditions (S_1_), but under synthetic air, it also supports nanowire nucleation and elongation.^45^ Therefore, the comparison between Sets S_1_ and S_2_ confirms that the presence of synthetic air is essential for high-density nanowire growth. Under ambient air, the Cu/Cu_2_O/CuO layers still form, but the limited oxygen availability and possible humidity effects inhibit nanowire development. Furthermore, Set S_1_ microtubes remain largely symmetric, whereas Set S_2_ structures often exhibit asymmetry, highlighting the role of the atmosphere in directing oxidation pathways.

#### Thermal Degradation of PU and Its Role in Cu Microwire Oxidation

As shown in Figure S5­(a–c) of Supporting Information, the polyurethane (PU) coating undergoes progressive thermal oxidative degradation that directly controls oxygen transport and copper oxidation. At 70–200 °C, PU degradation is initiated by a radical-chain oxidation mechanism, leading to chain scission and the formation of carbonyl-containing species. This stage produces limited mass loss, consistent with the plateau region in the TGA curve (Figure S5b).

At higher temperatures ≥250 °C, PU decomposition becomes dominant, resulting in the release of CO and CO_2_, as evidenced by the pronounced mass loss. The remaining carbonaceous residue forms a diffusion barrier, restricting the inward transport of oxygen toward the Cu core. Under these conditions, oxygen is preferentially consumed by carbon oxidation rather than direct copper oxidation.

Despite limited oxygen diffusion, Cu^+^ ions migrate outward toward the polymer/oxide interface, where they react with available oxygen. The imbalance between outward Cu^+^ diffusion and inward oxygen flux induces vacancy accumulation within the Cu core, leading to void formation and the development of a hollow structure via a Kirkendall-type mechanism, as schematically illustrated in Figure S5c.

### Proposed Mechanism for the Formation of Cu/Cu_2_O/CuO Microtubes and CuO Nanowires under Ambient and Synthetic Air Atmospheres

Figure 8 illustrates the proposed mechanism for the formation of hierarchical Cu/Cu_2_O/CuO semiconductor microtubes, synthesized via controlled thermal oxidation in either an ambient atmosphere or high-purity synthetic air. Based on experimental observations, we identify six sequential stages in the evolution of these microstructures:

**Figure 8:**
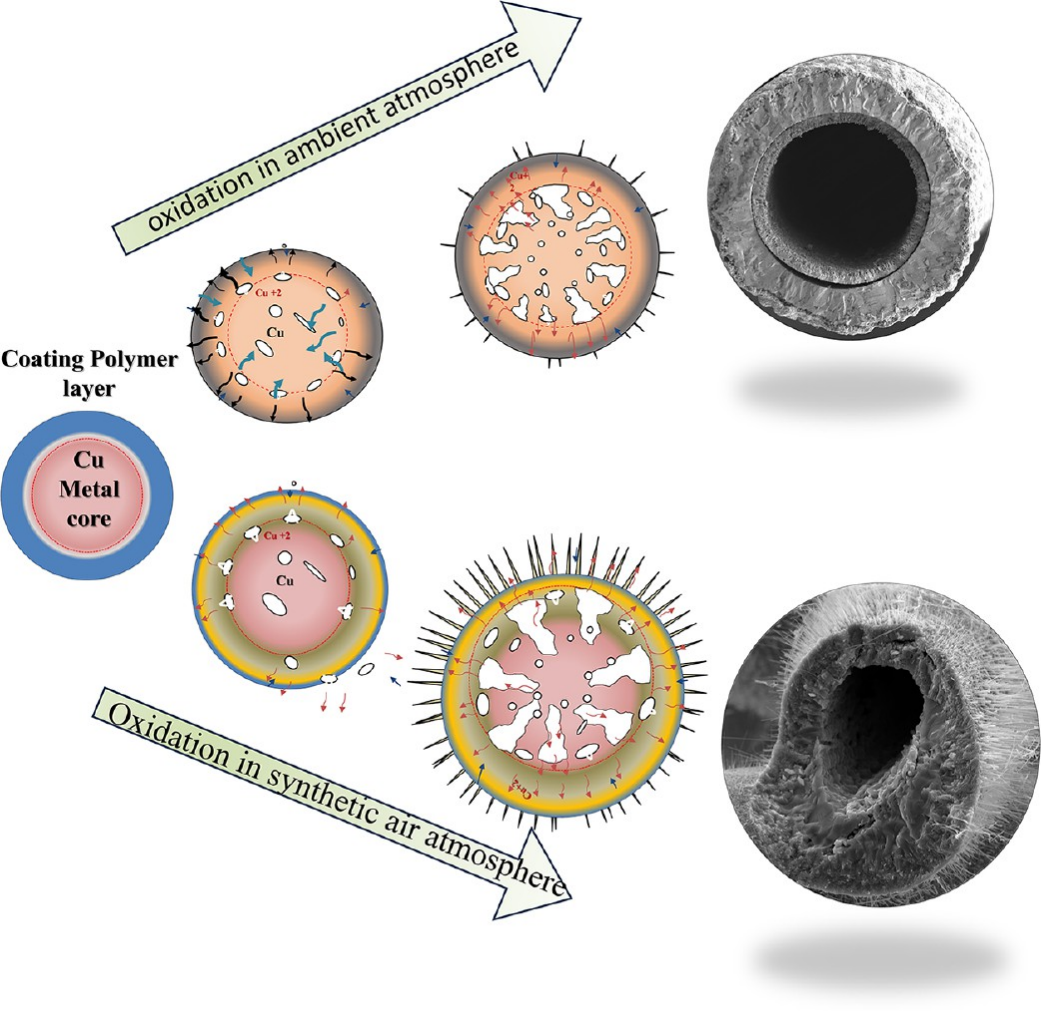
Proposed mechanism for Cu/Cu_2_O/CuO microtubule formation.

**Stage (i): Initial structure and polymer degradation.** The pristine copper microwire consists of a metallic Cu core encapsulated by a polyurethane (PU) coating that serves as a dielectric and mechanical barrier.^46^ As the temperature exceeded approximately 257 °C, the PU layer began to thermally degrade. Complete decomposition occurs around 400 °C, as confirmed by TGA analysis, leaving behind a thin carbonaceous residue (Cu@C or CuO@C). This residual carbon layer modulates oxygen diffusion, thereby influencing the subsequent oxidation kinetics.

**Stage (ii): Nucleation of oxide layers.** Following PU decomposition, the exposed Cu surface reacts with atmospheric oxygen, initiating the formation of a thin Cu_2_O interfacial layer, which progressively transforms into CuO on the outermost surface.^47^ The residual carbon film acts as a partial diffusion barrier, enabling a controlled and gradual oxidation process.

**Stage (iii): Development of diffusion gradients and vacancy formation (Kirkendall effect).** As oxidation advances, pronounced chemical and thermal gradients emerge between the metallic Cu core and the surrounding Cu_2_O/CuO shells.^48^ The outward diffusion of Cu^+^ ions toward the surface outpaces the inward diffusion of oxygen, resulting in a flux imbalance (*J*_Cu_ > *J*_O_). This disparity leads to a net inward flow of vacancies, consistent with the classical Kirkendall mechanism. The coalescence of these vacancies within the metallic core initiates internal void formation.^49^

**Stage (iv): Pore coalescence and structural collapse.** With continued oxidation, the accumulation and coalescence of vacancies generate microvoids that progressively expand within the Cu core. The associated mechanical stresses promote the growth and interconnection of these pores, leading to a partial structural collapse and hollowing of the metallic interior.

**Stage (v): Formation of hollow microtubes.** As the copper reservoir is depleted and micropores coalesce, the microwire fully transforms into a hollow Cu/Cu_2_O/CuO microtube.^49^ Concentric oxide layers develop as a result of the sustained ionic diffusion and chemical potential gradients. The complete hollowing process typically occurs within approximately six h, indicating the high ionic mobility and accelerated oxidation kinetics characteristic of this system.

**Stage (vi): Nanowire growth under synthetic air.** When oxidation takes place under a continuous flow of synthetic air (O_2_/N_2_ mixture), the outer CuO layer becomes highly oxidized and structurally favorable for nanowire nucleation. This oxygen-rich, dry, and chemically stable atmosphere promotes vertical CuO nanowire growth through three concurrent processes: (a) diffusion of Cu^+^ ions along twin boundaries in CuO grains, which act as preferential, low-energy pathways for anisotropic growth; (b) surface diffusion of Cu^+^ toward reactive oxygen sites, enabling localized nucleation events; and (c) maintenance of a chemical potential gradient between the Cu-rich interior and the O_2_-rich surface, driving unidirectional ion transport that sustains axial elongation.^40^

The morphological asymmetry observed under synthetic air conditions cannot be attributed solely to the oxygen concentration. In the TGA configuration, the presence of a controlled and directional gas flow is expected to enhance asymmetric mass transport, promoting preferential outward Cu^+^ diffusion and localized oxidation. In contrast, oxidation under ambient air in a muffle furnace occurs under less defined flow conditions, resulting in more isotropic oxidation behavior

## Conclusions

This work demonstrates that the thermal oxidation of polymer-coated copper microwires enables the controlled formation of hierarchical Cu/Cu_2_O/CuO hollow microtubes. The thermal degradation of the polyurethane coating produces a thin carbonaceous interlayer that modulates oxygen diffusion and promotes the preferential outward migration of the Cu^+^/Cu^2+^ ions. The resulting unbalanced ionic flux drives vacancy coalescence and hollowing through a Kirkendall-type mechanism.

The process reveals enhanced ionic transport in polymer-assisted oxidation, where the synergy between oxidation kinetics and carbon-mediated diffusion accelerates the cation mobility and void formation. Under synthetic air, enhanced oxygen activity favors the growth of vertically aligned CuO nanowires up to 20 μm long, driven by surface diffusion and twin-boundary-mediated ion migration. Although the present results highlight the role of oxygen availability in anisotropic oxidation, further investigation is needed to elucidate the relative contributions of the oxygen partial pressure and ambient humidity on oxidation kinetics and nanowire growth.

At synthetic air flow rates above 100 mL·min^–1^, an increase in oxygen flux is expected, leading to faster oxidation kinetics and enhanced directional mass transport. While higher flow rates may accelerate Cu oxidation and vacancy injection, excessively high oxygen availability, such as above a certain threshold, may reduce diffusion-limited growth conditions, potentially suppressing the controlled formation of CuO nanowires and promoting the growth of denser oxide shells. Therefore, the chosen flow rate represents a balance between providing a sufficient oxygen supply and maintaining the necessary asymmetric diffusion conditions for the formation of hierarchical microtubes and nanowires.

This simple and scalable approach enables the conversion of e-waste Cu microwires into functional hierarchical oxides, integrating semiconducting and potentially magnetic phases within a single architecture. The resulting CuO nanowire-decorated microtubes offer a versatile platform for next-generation devices in sensing, catalysis, and nanoelectronics and provide new insights into colossal ion diffusion and controlled Kirkendall transformations in metal–oxide systems.

## Experimental Section

### Synthesis of Hierarchical Copper Oxide Microstructures

Hollow copper oxide microtubes and hierarchical microstructures decorated with CuO nanowires were synthesized via the thermal oxidation of metallic copper (Cu) microwires at elevated temperatures. This approach was inspired by processes previously reported for the formation of hollow and hierarchical nanostructures, such as ZnO, TiO_2_, and FeO microtubes.^5–8^ Recycled copper wires sourced from commercial headphones were employed as precursors. The copper purity was determined to be 99.7% by neutron activation analysis (NAA), performed in the IEA-R1 nuclear reactor (IPEN, Brazil). For this purpose, 10 mg of each sample was irradiated for 3 s under a thermal neutron flux of approximately 3.1 × 10^12^ n·cm^–2^·s^–1^. The thermal oxidation process was conducted in two sequential steps. First, the samples were heated at a rate of 20 °C·min^–1^ up to 400 °C and held for 10 min. Next, the temperature was increased to 600 °C at the same heating rate and maintained for 6 h, before being allowed to cool naturally inside the furnace. Two distinct sample sets were processed under different atmospheric conditions using copper wires from identical headphone cable models: (a) Sample Set 1 (S_1_): Cu wires were placed in 14 mL porcelain crucibles (30 mm height) and oxidized in a conventional muffle furnace (Spencer) under ambient atmospheric conditions (relative humidity ∼70%). (b) Sample Set 2 (S_2_): Cu wires were placed in alumina crucibles and subjected to thermal treatment in a Shimadzu DTG-60 thermogravimetric analyzer under a controlled flow of high-purity synthetic air (100 mL·min^–1^). Figure 9 schematically illustrates the process of sample formation and characterization.

**Figure 9:**
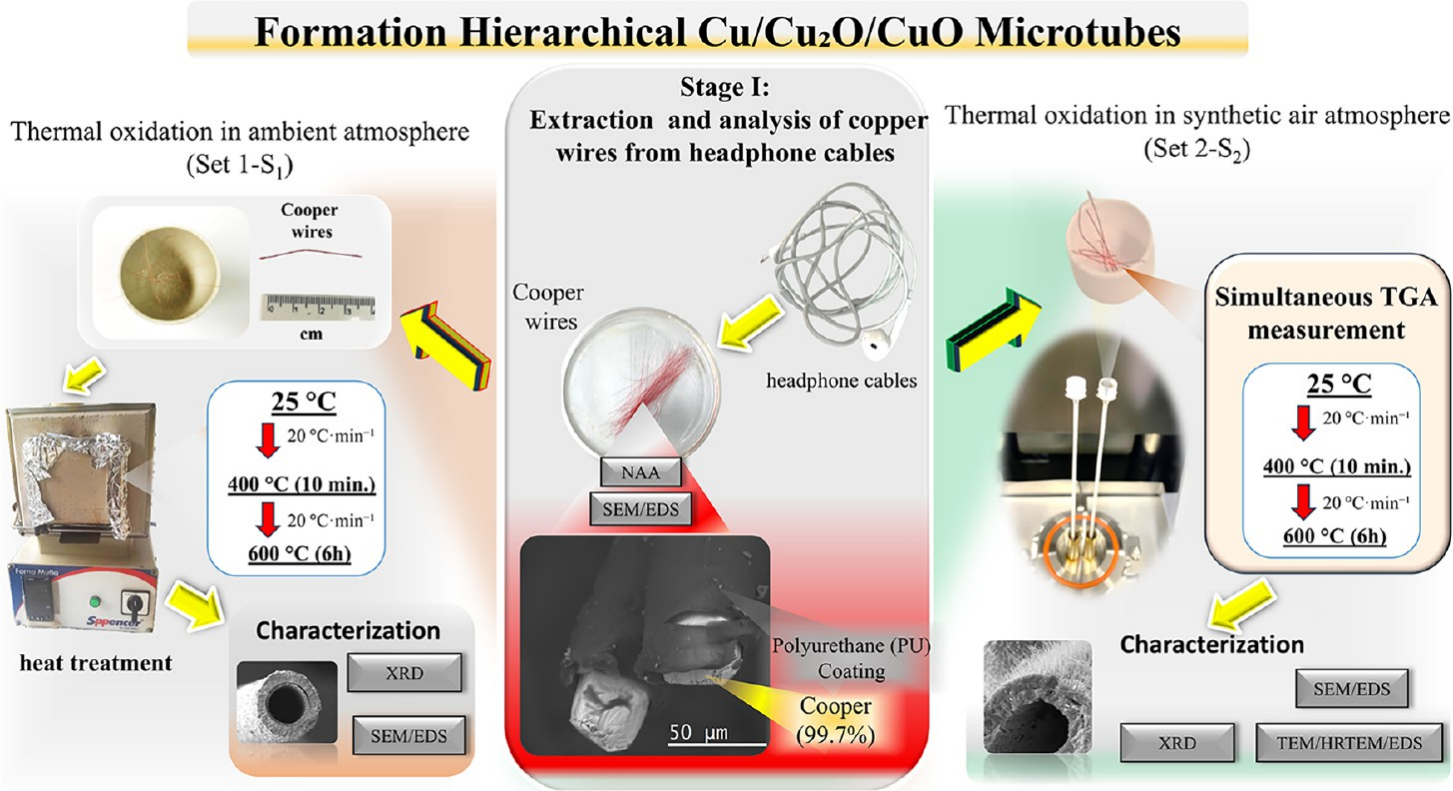
Schematic representation of the preparation and characterization steps of hierarchical microtube structures under different atmospheres, highlighting the synthesis conditions and materials employed. The characterization focuses on techniques such as SEM-EDS, XRD, HRTEM, and TGA.

## Supplementary Material



## Notes

FTIR spectrum evidencing the PU coating on the Cu microwires; SEM micrographs of S_1A_ and S_2A_; Raman spectra for S_1B_; and SEM image and thermogravimetric analysis (TGA) of a PU-coated Cu microwire after thermal treatment (PDF)
