# Supplementary material for: Atmosphere-Controlled Synthesis of Hierarchical Cu/Cu2O/CuO Microtube Architectures Decorated with High-Density CuO Nanowires from Recycled E‑Waste
Source: Langmuir. 2026 Mar 20;42(13):9330–41. doi: 10.1021/acs.langmuir.5c06745 (PMC13063809; doi:10.1021/acs.langmuir.5c06745)
Supplement: Supplementary file 1 [file la5c06745_si_001.pdf]

Supporting Information

for

Atmosphere-Controlled Synthesis of Hierarchical $Cu/Cu_2O/CuO$ Microtube Architectures Decorated with High-Density CuO Nanowires from Recycled E-Waste

Suzilene V. Santos,[†] Crystian W. C. Silva,[‡] Pedro H. Britto-Costa,[¶] Thiago Lopes,[§] Sávio F. Silva,[†] Cleidilane S. Costa,[†] Larissa Otubo,[‡] C. M. Rivaldo-Gómez,[‖] Artur W. Carbonari,[*,‡,⊥] and Gabriel A. Cabrera-Pasca[*,§,†]

†Universidade Federal do Pará (UFPA), Abaetetuba – Pará, 09210-580, Brazil

‡Instituto de Pesquisas Energéticas e Nucleares, Comissão Nacional de Energia Nuclear, IPEN-CNEN/SP, São Paulo, 05508-000, SP, Brazil

¶Research Centre for Greenhouse Gas Innovation (RCGI), University of São Paulo, Escola Politécnica, Av. Professor Mello Moraes, 2231 – Cidade Universitária, São Paulo - SP, 05508-030, Brazil

§Research Center for Greenhouse Gas Innovation (RCGI), University of São Paulo, Escola Politécnica, Av. Professor Mello Moraes, 2231 – Cidade Universitária, São Paulo - SP, 05508-030, Brazil

‖Research Center for Greenhouse Gas Innovation (RCGI), University of São Paulo, Escola Politécnica, Av. Professor Mello Moraes, 2231 – Cidade Universitária, São Paulo - SP, 05508-030, Brazil

⊥European Organization for Nuclear Research, Esplanade des Particules 1, 1217 Meyrin, Switzerland

E-mail: carbonar@ipen.br; gpasca@ufpa.br

Contents

- Figures S1 to S5

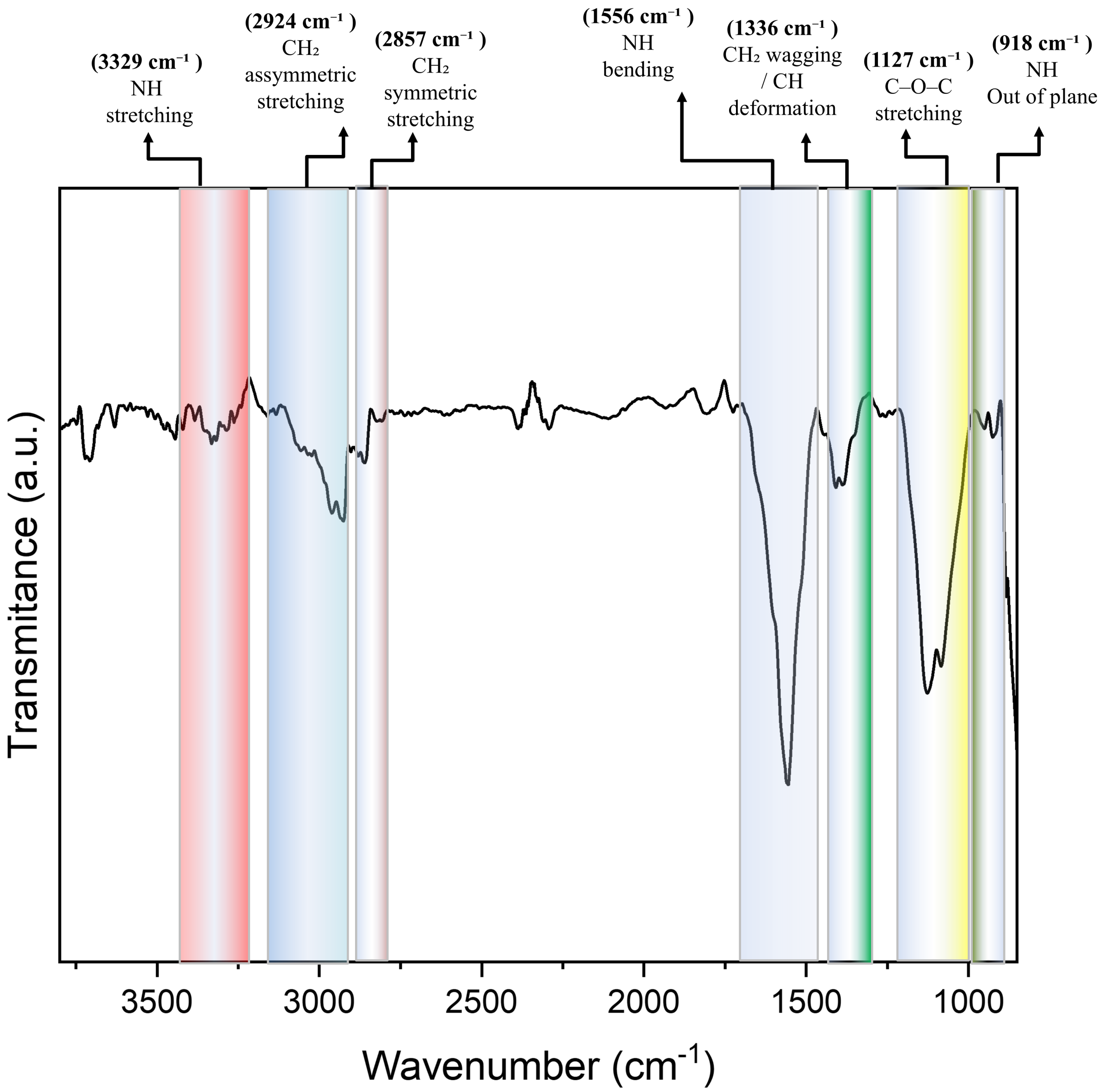

Figure S1: FTIR spectrum evidencing the PU coating on the Cu microwires

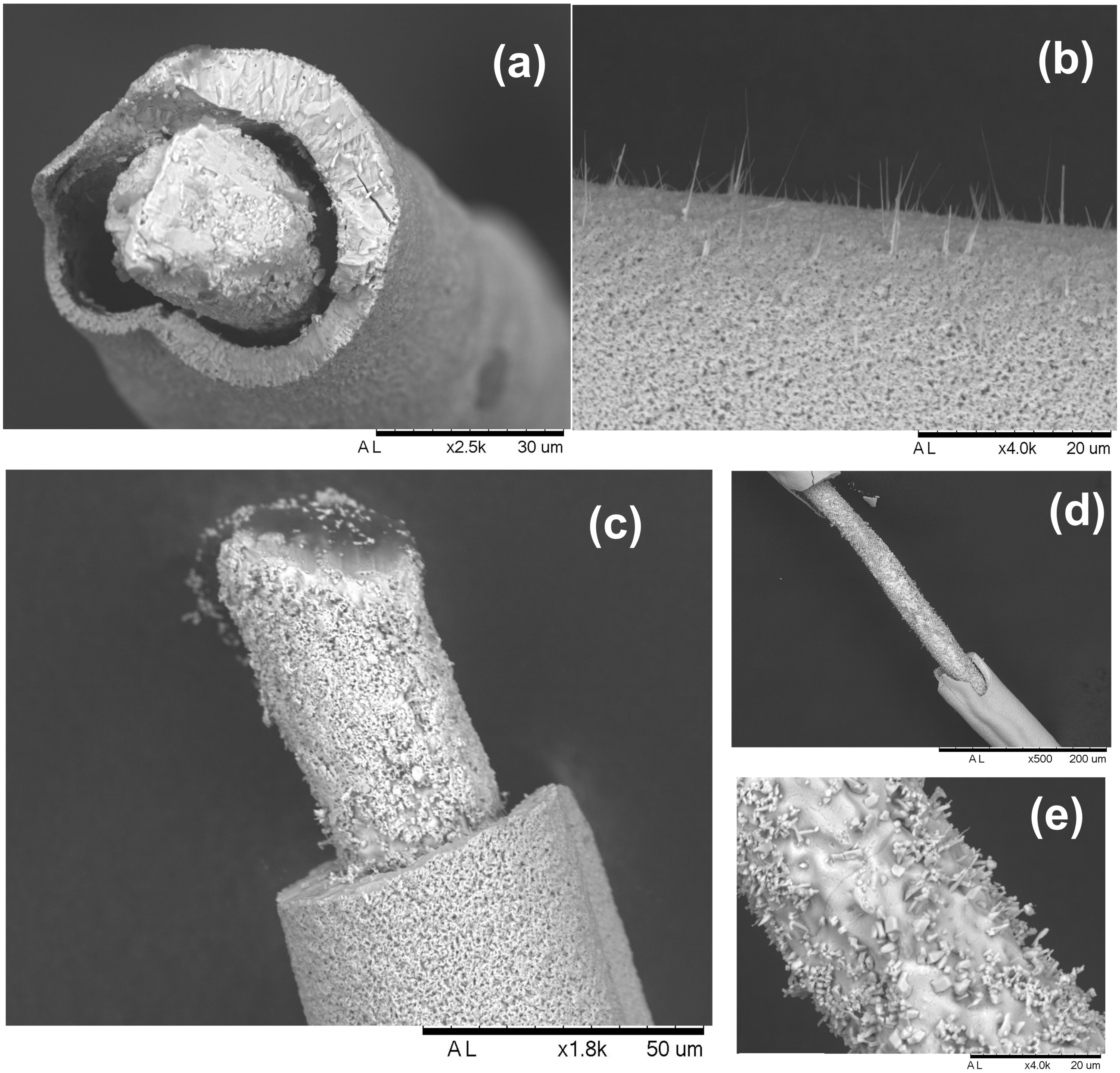

Figure S2: SEM micrographs of S_{1A} (600 °C for 1 hour in a conventional muffle furnace, followed by cooling in air): (a) frontal view of the microtube; (b) microtube surface showing some nanowires; (c–e) metallic copper core at the center of the samples.

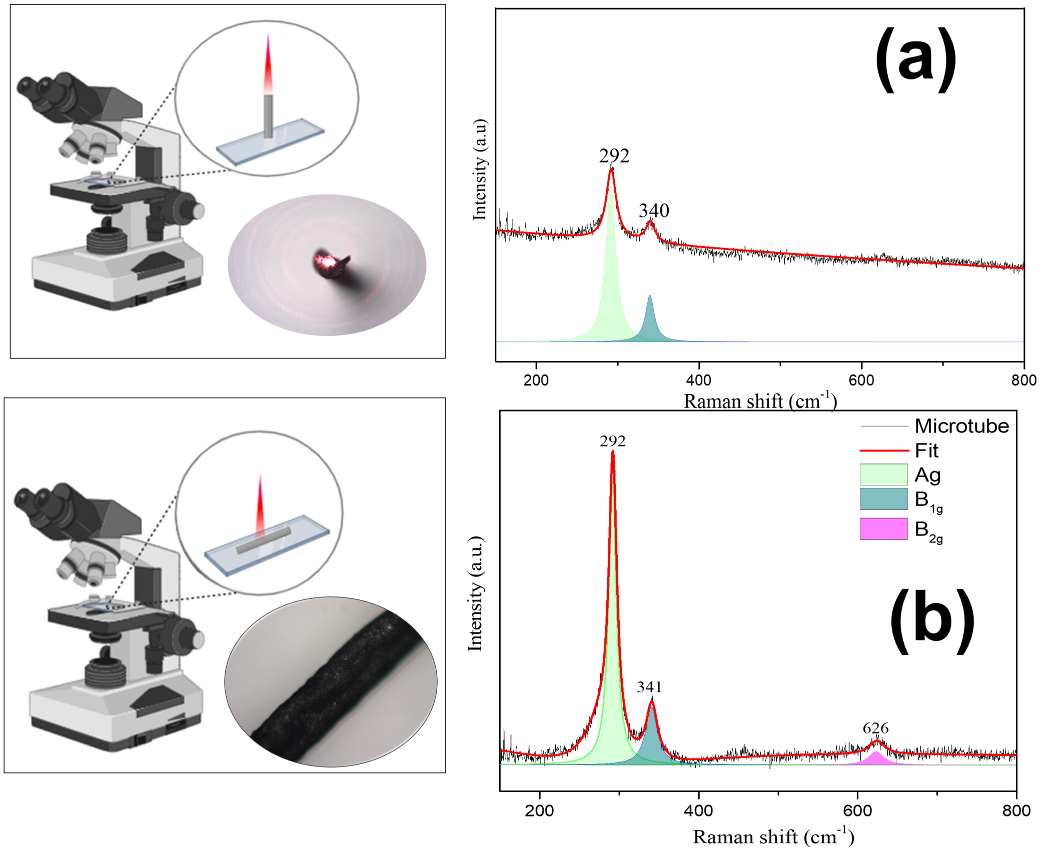

Figure S3: Raman spectra for S_{1B} (600 °C for 6 hours in ambient atmosphere,in a conventional muffle furnace, followed by cooling in air): (a) spectrum of the vertical section of the microtubes, (b) spectrum of the horizontal section.

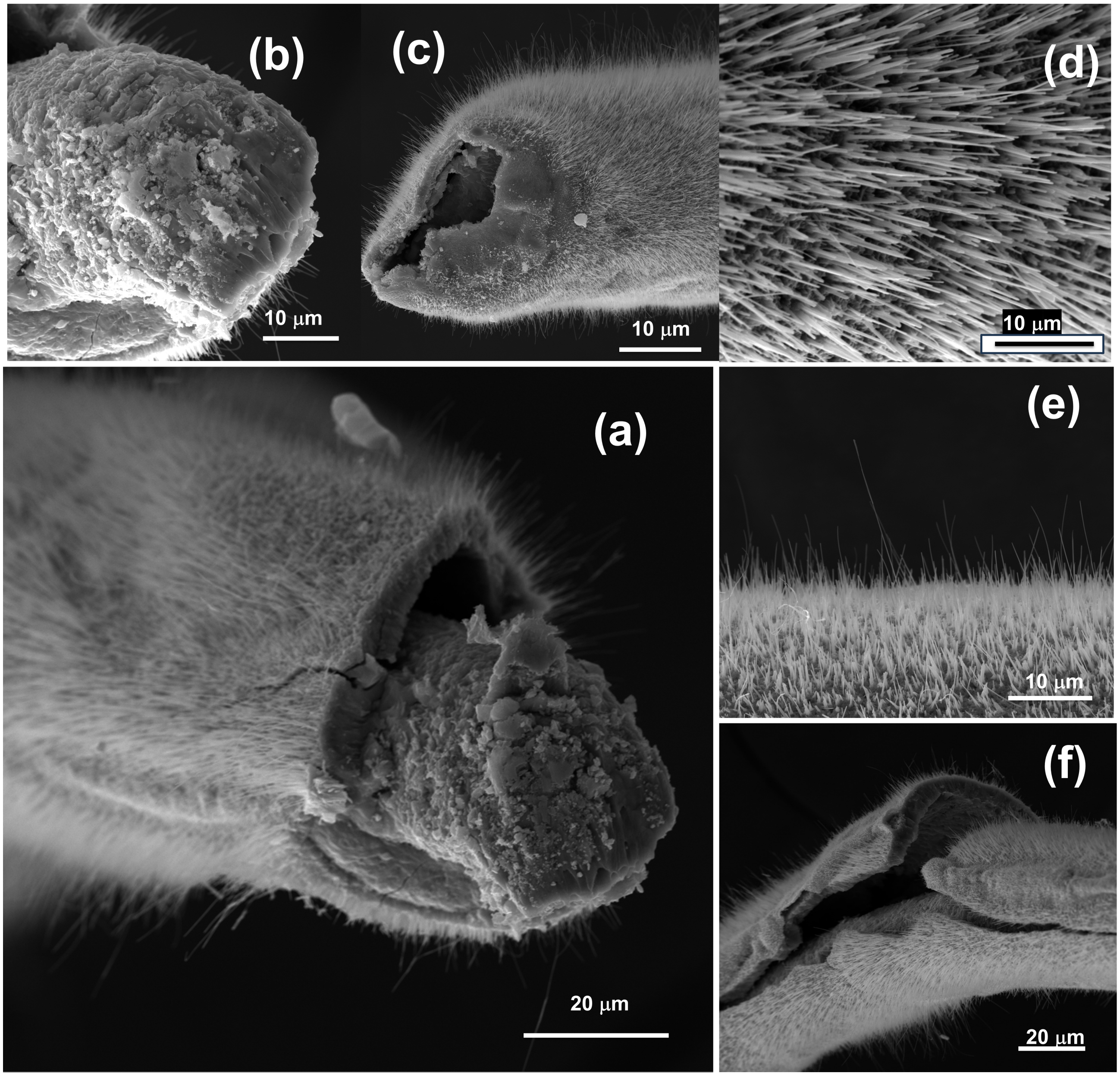

Figure S4: SEM micrographs of S_{2A} (600 °C for 5 minutes in synthetic air): (a) microtube with several nanowires on the surface, with residual metallic copper at the center; (b–f) surface views decorated with nanowires.

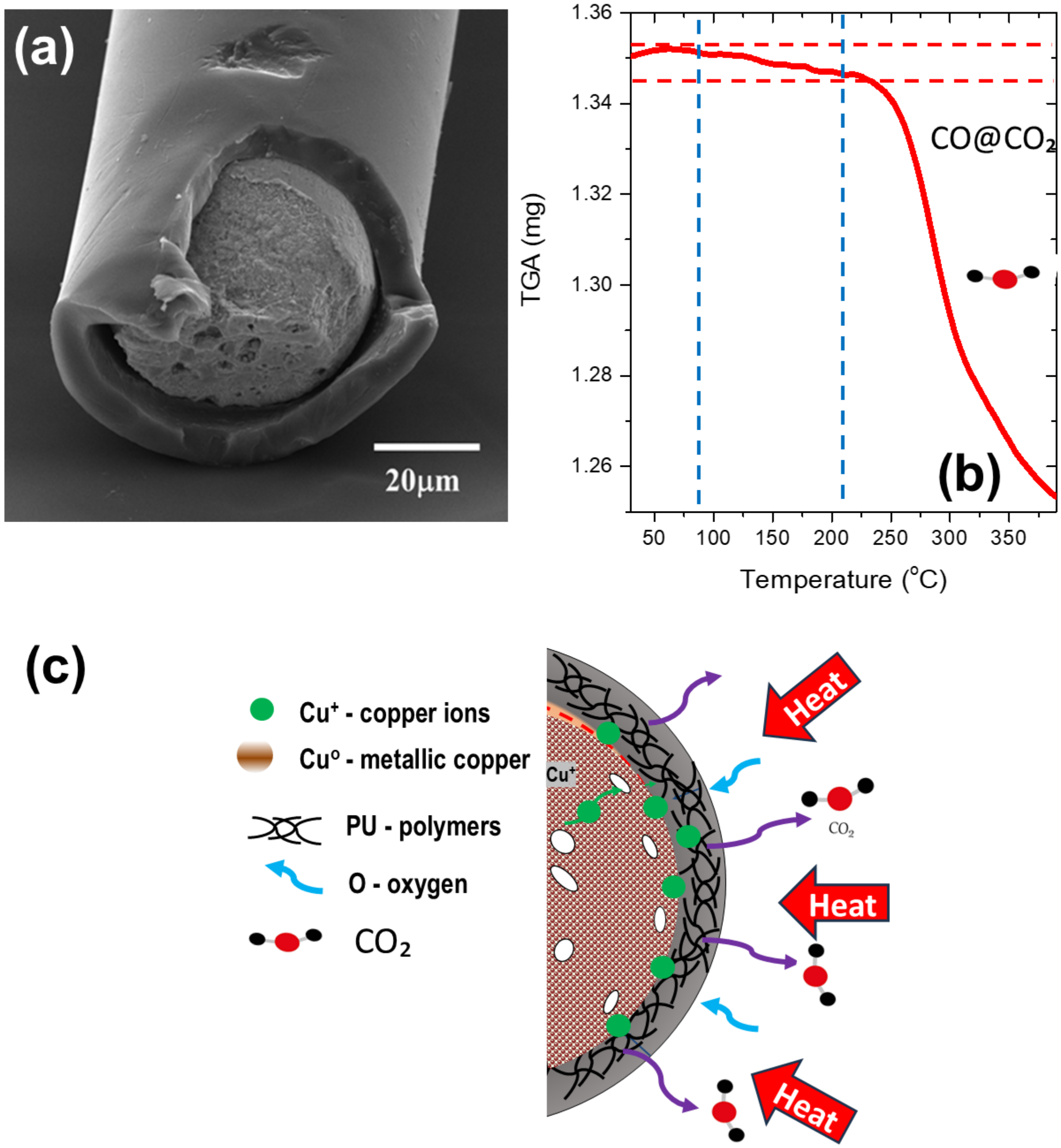

Figure S5: (a) SEM image of a PU-coated Cu microwire after thermal treatment, showing the formation of a hollow structure. (b) Thermogravimetric analysis (TGA) of the PU-coated microwires, indicating a low-temperature oxidative degradation regime (∼70–200 °C) followed by rapid mass loss associated with polymer decomposition and CO/CO_2 release at higher temperatures. (c) Schematic illustration of the proposed mechanism, where PU degradation and residual carbon limit oxygen diffusion, while outward Cu^+ migration dominates, leading to vacancy accumulation, void formation, and hollow microtube development via a Kirkendall-type process.
